# Morphological Substrates for Atrial Arrhythmogenesis in a Heart With Atrioventricular Septal Defect

**DOI:** 10.3389/fphys.2018.01071

**Published:** 2018-08-23

**Authors:** Robert S. Stephenson, Jack Rowley-Nobel, Caroline B. Jones, Rafael Guerrero, Tristan Lowe, Jichao Zhao, Henggui Zhang, Jonathan C. Jarvis

**Affiliations:** ^1^Comparative Medicine Lab, Institute of Clinical Medicine, Aarhus University, Aarhus, Denmark; ^2^School of Physics and Astronomy, University of Manchester, Manchester, United Kingdom; ^3^Department of Cardiology, Alder Hey Children's Hospital, Liverpool, United Kingdom; ^4^Department of Cardiac Surgery, Alder Hey Children's Hospital, Liverpool, United Kingdom; ^5^Manchester X-ray Imaging Facility, Photon Science Institute, University of Manchester, Manchester, United Kingdom; ^6^Auckland Bioengineering Institute, Auckland University, Auckland, New Zealand; ^7^School of Sport and Exercise Sciences, Liverpool John Moores University, Liverpool, United Kingdom

**Keywords:** arrhythmias cardiac, atrial fibrillation (AF), re-entry, micro-computed tomography, mathematical modeling, myocyte orientation, congenital heart disease (CHD), atrioventricular septal defect

## Abstract

Due to advances in corrective surgery, congenital heart disease has an ever growing patient population. Atrial arrhythmias are frequently observed pre- and post-surgical correction. Pharmaceutical antiarrhythmic therapy is not always effective, therefore many symptomatic patients undergo catheter ablation therapy. In patients with atrioventricular septal defects (AVSD), ablation therapy itself has mixed success; arrhythmogenic recurrences are common, and because of the anatomical displacement of the atrioventricular node, 3-degree heart block post-ablation is a real concern. In order to develop optimal and safe ablation strategies, the field of congenital cardiac electrophysiology must combine knowledge from clinical electrophysiology with a thorough understanding of the anatomical substrates for arrhythmias. Using image-based analysis and multi-cellular mathematical modeling of electrical activation, we show how the anatomical alterations characteristic of an AVSD serve as arrhythmogenic substrates. Using *ex-vivo* contrast enhanced micro-computed tomography we imaged post-mortem the heart of a 5 month old male with AVSD at an isometric spatial resolution of 38 μm. Morphological analysis revealed the 3D disposition of the cardiac conduction system for the first time in an intact heart with this human congenital malformation. We observed displacement of the compact atrioventricular node inferiorly to the ostium of the coronary sinus. Myocyte orientation analysis revealed that the normal arrangement of the major atrial muscle bundles was preserved but was modified in the septal region. Models of electrical activation suggest the disposition of the myocytes within the atrial muscle bundles associated with the “fast pathway,” together with the displaced atrioventricular node, serve as potential substrates for re-entry and possibly atrial fibrillation. This study used archived human hearts, showing them to be a valuable resource for the mathematical modeling community, and opening new possibilities for the investigations of arrhythmogenesis and ablation strategies in the congenitally malformed heart.

## Introduction

**Figure 1:**
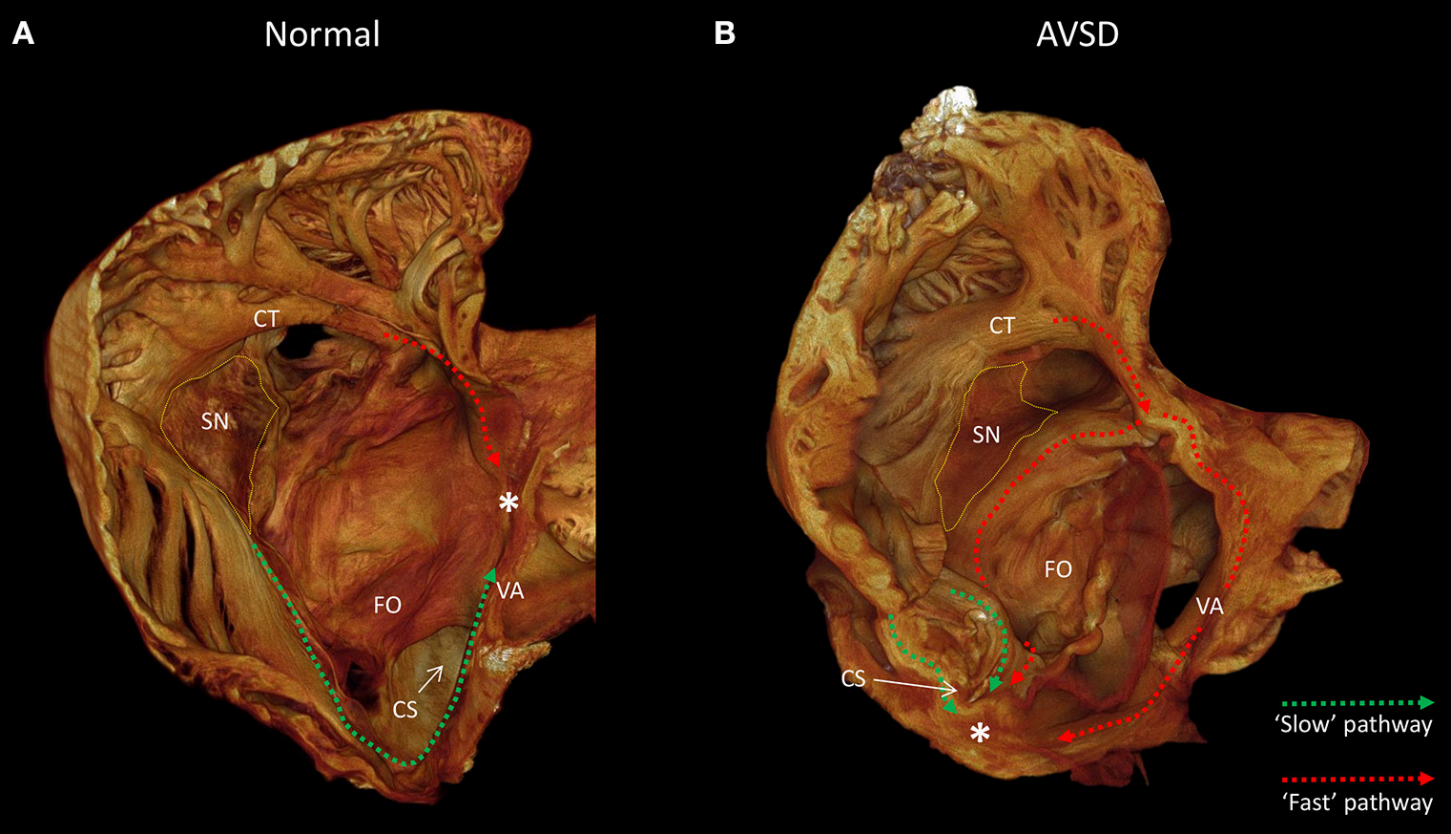
The anatomy of the slow and fast pathways in the right atrium. **(A)** indicates the anatomical locations of the slow pathway (green), fast pathway (red), and compact atrioventricular node (*) in the normal heart, viewed from inferior-lateral position. **(B)** indicates the hypothesized anatomical location of the slow pathway (green) and fast pathway (red) in a heart with a atrioventricular septal defect, note the compact atrioventricular node (*) is displaced posterior-inferiorly. Images derived from micro-CT data. *Location of compact atrioventricular node; CS, coronary sinus; CT, terminal crest; FO, fossa ovale; SN, sinus node; VA, valve annulus.

The competency and success of corrective surgery is ever improving, as a result congenital heart disease has an ever growing patient population, with adults now outnumbering children (Khairy, 2008). Despite this, atrial arrhythmias are frequently observed pre- and post-surgical correction. Patients with atrioventricular septal defect (AVSD) or atrioventricular canal defect (AVCD) have a common atrioventricular connection, this occurs due to incorrect fusion of the endocardial cushions with the atrial septum and muscular ventricular septum (Anderson et al., 2000, 2008). Preoperative electrophysiological studies of AVSD patients have shown cases of atrioventricular re-entrant tachycardia (Khairy et al., 2007), atrial fibrillation (Daliento et al., 1991; Khairy et al., 2006) and supra-Hisian first degree AV block, and confirm inter-nodal conduction delay in the majority of patients (Fournier et al., 1986). Persistent AV block is present in up to 7% of patients in the immediate post-operative period and approximately 2% on follow up (Daliento et al., 1991; Boening et al., 2002), with atrial fibrillation or flutter noted in 5% of patients after surgical repair (Vetter and Horowitz, 1982; Daliento et al., 1991). Many symptomatic patients undergo catheter ablation therapy with varying success, arrhythmogenic recurrences are common. During ablation therapy the interventional cardiologist will target the major muscle bundles believed to be responsible for the inter-nodal conduction disturbance. These bundles have been described previously based on their anatomical appearance and the alignment of the myocyte chains within them (James, 1963; Merideth and Titus, 1968; Sanchez-Quintana et al., 1997). More recently these pathways have been described based on their electrophysiology using optical mapping, and are described in the context of the so-called dual pathway physiology (Hucker et al., 2008; Mani and Pavri, 2014; George et al., 2017). The pathways are termed the “slow” and “fast” pathways; in the healthy heart the “fast” pathway is the dominant conduction pathway between the sinus node and atrioventricular node. Anatomically the fast pathway courses the anterior-superior aspect of the inter-atrial septum and is associated proximally with the terminal crest and distally with the transitional cells surrounding the compact AV node [(Mani and Pavri, 2014; George et al., 2017); Figure 1A]. Conversely, the “slow” pathway has a less direct course, it runs inferior to the coronary sinus ostium and fossa ovale, and is associated with the flutter isthmus and the inferior nodal extension. In AVSD the atrioventricular node is displaced. The compact atrioventricular node no longer lies at the apex of the triangle of Koch (Figure 1A), but in a posterior-inferior position, anterior to the ostium of the coronary sinus at the point where the posterior-inferior rims of muscular ventricular and atrial septa join [(Moorman et al., 1998); Figure 1B]. This inevitably changes the anatomical course of the “fast” and “slow” pathways (Figure 1B). Conduction disturbances in AVSD patients are associated with prolonged inter-nodal conduction times and numerous conduction disturbances (Waldo et al., 1973; Jacobsen et al., 1976; Fournier et al., 1986; Khairy et al., 2006), presumably because the inter-nodal muscle bundles are distorted or modified as they course the atria (Waldo et al., 1973).

Inter-nodal conduction is thus dictated by the location of the nodal tissues and the muscle bundles connecting them. In order to develop optimal and safe ablation strategies for congenitally malformed hearts, the field of congenital cardiac electrophysiology requires an integration of clinical electrophysiology with a thorough understanding of the anatomical substrates for arrhythmias. Guided by the available clinical electrophysiological data we hypothesize that anatomical displacement of the compact atrioventricular node and modification of the dual pathway physiology act as substrates for arrhythmogenesis in AVSD patients. We use image data acquired by micro-computed tomography (micro-CT), as described previously (Stephenson et al., 2012, 2017), to extract myocyte orientation and to identify the 3D disposition of the nodal tissue for the first time in an intact heart with AVSD. This information is then incorporated into electrophysiologically accurate mathematical models of electrical activation to assess the influence of these anatomical alterations on inter-nodal conduction. This study also demonstrates the suitability of long term stored archived human hearts as a resource for the mathematical modeling community in investigations of arrhythmogenesis in the congenitally malformed heart.

## Methods

### Ethical approval statement

We obtained NHS ethical approval to scan congenitally malformed samples from the Alder Hey archive in Liverpool UK. Samples had been consented and placed in the archive in the 1970s.

### Sample preparation

We chose a sample from the archive free of clotted blood, and probably perfused via the coronary circulation prior to fixation. The sample was from a male who died aged 5 months, and has been in storage for approximately 50 years since the 1970s. The heart dimensions; max length ~70 mm, max width ~55 mm. The sample was prepared for scanning by immersion in 3.75% iodine/potassium iodide (I_2_KI) in PBFS for 2 weeks, refreshing the solution at 1 week (Stephenson et al., 2017). Iodine molecules are progressively and differentially absorbed by the tissues, permitting discrimination of fat, working myocardium, conducting tissues, and fibrous tissue.

### Scanning

The sample was scanned in the Nikon Metris XTEK 320 kV Custom Bay at the Manchester X-Ray Imaging Facility, University of Manchester, as previously described by Stephenson et al. (2012, 2017). Prior to scanning the sample was drained and rinsed in saline to remove excess contrast agent. Plastic wrap provided containment of the tissue, and maintained the original shape of the sample. The heart was immobilized in a plastic tube to reduce movement during the imaging process. Scans were acquired with an X-ray energy of ~95 kV. 360° scans were performed and data was collected from 3142 projections. A tungsten target was used for all scans, with a 0.25 mm aluminum filter. Total scan times were approximately 50 min. Data was reconstructed using filtered back-projection, resulting in tomographic image data with an isotropic voxel size of 38.5 × 38.5 × 38.5 μm. After scanning, the sample was placed back in to formaldehyde solution to allow passive removal of the iodine.

### Image analysis

The datasets were examined using Amira (5.3.3) and analyzed using objective semi-automatic segmentation methods as described previously (Jarvis and Stephenson, 2013; Stephenson et al., 2017). Muscle bundles associated with the slow and fast pathways along with the terminal crest and common valve annulus were segmented based on the ability to visualize and trace the longitudinal chains of myocyte in the individual muscle bundles using the micro-CT image data. The specialized tissues of the cardiac conduction system were segmented based on their differential attenuation. The electrophysiological block zone, a region of slow conductance between the sinus node and atrial septum, was subjectively placed based on previous representations (Boyett et al., 2000). Myocyte orientation was extracted from the micro-CT data using eigen analysis of the 3D structure tensor as described previously (Ni et al., 2013). To generate myocyte orientation files the raw data was first down-sampled to a spatial resolution of 0.15 mm.

### Modeling

To generate a geometrical model for the modeling protocols the raw data was down-sampled to an isotropic spatial resolution of 0.15 mm, which is close to the length of atrial myocytes. Virtual suturing of the dissected borders was performed prior to modeling, such regions were assigned atrial electrophysiological characteristics. Muscle bundle and whole atria electrical activation was modeled using the Coleman-Ni-Zhang (CNZ) model (Ni et al., 2017). In this study cells of the conduction system and the segmented muscle bundles were all assigned as “CT” type. The cells of the atrial working myocardium were assigned as “RA” type. Cells in the region labeled as the “block zone” were assigned as “RA” type but with reduced excitability, this was achieved by reducing their calcium and sodium conductance to 50%. The diffusion parameters were set to a ratio of 8:1 (along the myocyte chain:perpendicular to the myocyte chain). Diffusion coefficients and spatial resolution gave a conduction velocity of 68.2 cm/s for the RA cells. This is within the range of (70.2 ± 9.9) cm/s measured experimentally in RA cells (Kojodjojo et al., 2006). A series of external stimuli with an amplitude of 20 pA/pF and a duration of 2 ms were applied to the sinus node cells in the standard protocols. At fast pacing rates, stimuli with an amplitude of 40 pA/pF and a duration of 4 ms were implemented. During the pacing protocols various S1-S2 intervals were investigated, these ranged from 250 to 400 ms.

## Results

### Morphological analysis by micro-computed tomography

**Figure 2:**
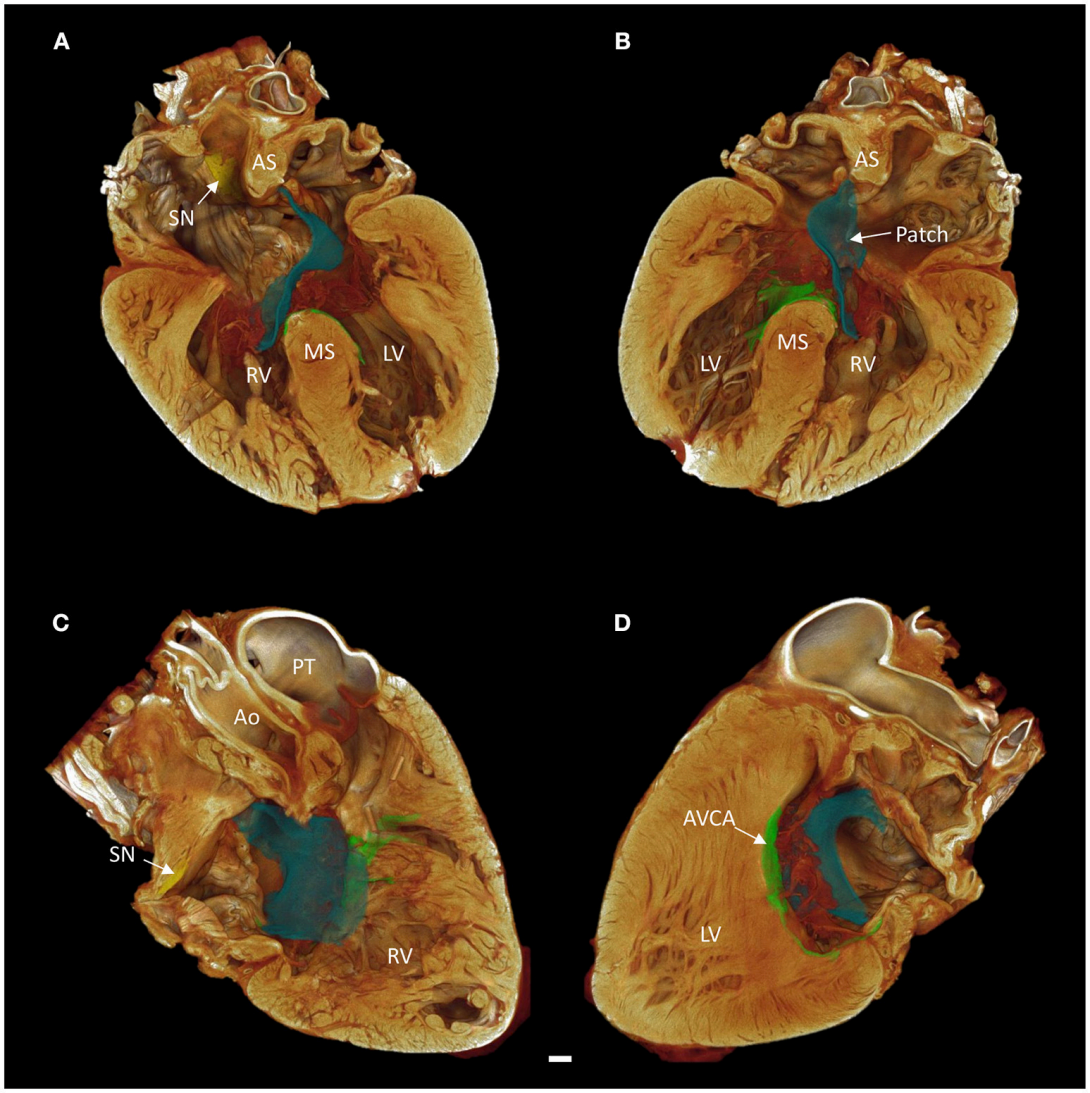
Long axis volume renderings of a heart with atrioventricular septal defect (AVSD). **(A)** Anterior 4-chamber view, **(B)** posterior 4-chamber view, **(C)** right side two-chamber view, **(D)** left side two-chamber view. The sinus node is shown in yellow, the atrioventricular conduction axis in green, and the surgical patch in blue. Images derived from micro-CT data. Ao, aorta; AS, atrial septum; AVCA, atrioventricular conduction axis; LV, left ventricle; MS, muscular ventricular septum; PT, pulmonary trunk; RV, right ventricle; SN, sinus node. Scale bar represents 3 mm.

The contrast enhanced micro-CT data allowed fast and unequivocal classification of the congenital malformation. We confirmed the heart to have an atrioventricular septal defect with common atrioventricular junction and aligned atrial and muscular ventricular septa (Figure 2).

**Figure 3:**
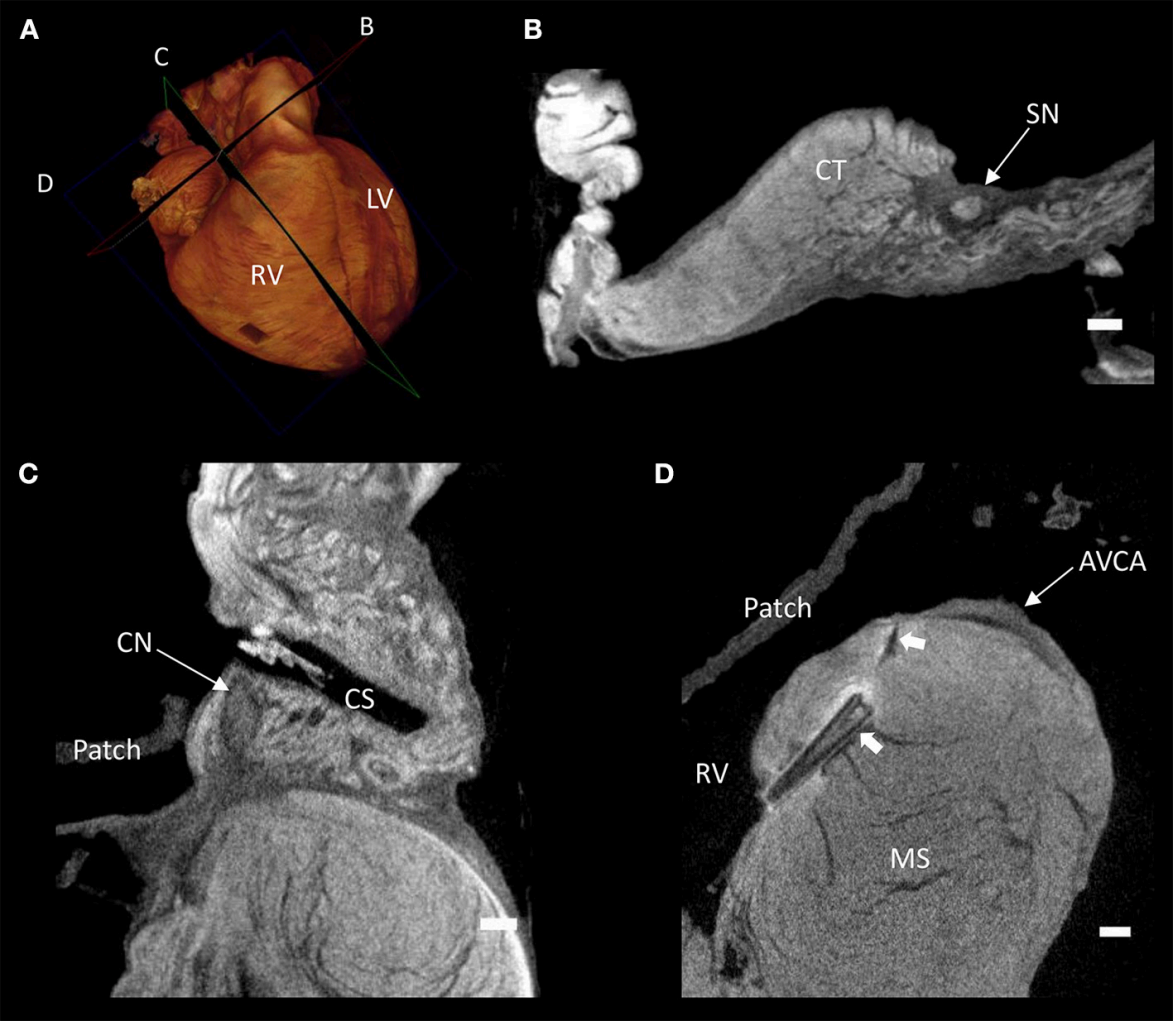
Virtual histology of the cardiac conduction system in a heart with atrioventricular septal defect (AVSD). **(A)** Volume rendering of the whole heart illustrating the virtual cutting planes used in **(B–D)**. **(B)** Short axis micro-CT section of the sinus node, **(C)** two-chamber micro-CT section of the compact atrioventricular node, **(D)** 4-chamber micro-CT section of the atrioventricular conduction axis. AVCA, atrioventricular conduction axis; CN, compact atrioventricular node; CS, coronary sinus; CT, terminal crest; LV, left ventricle; MS, muscular ventricular septum; RV, right ventricle; SN, sinus node; solid arrow heads- pledget and suture line. Scale bars represents 1 mm.

**Figure 4:**
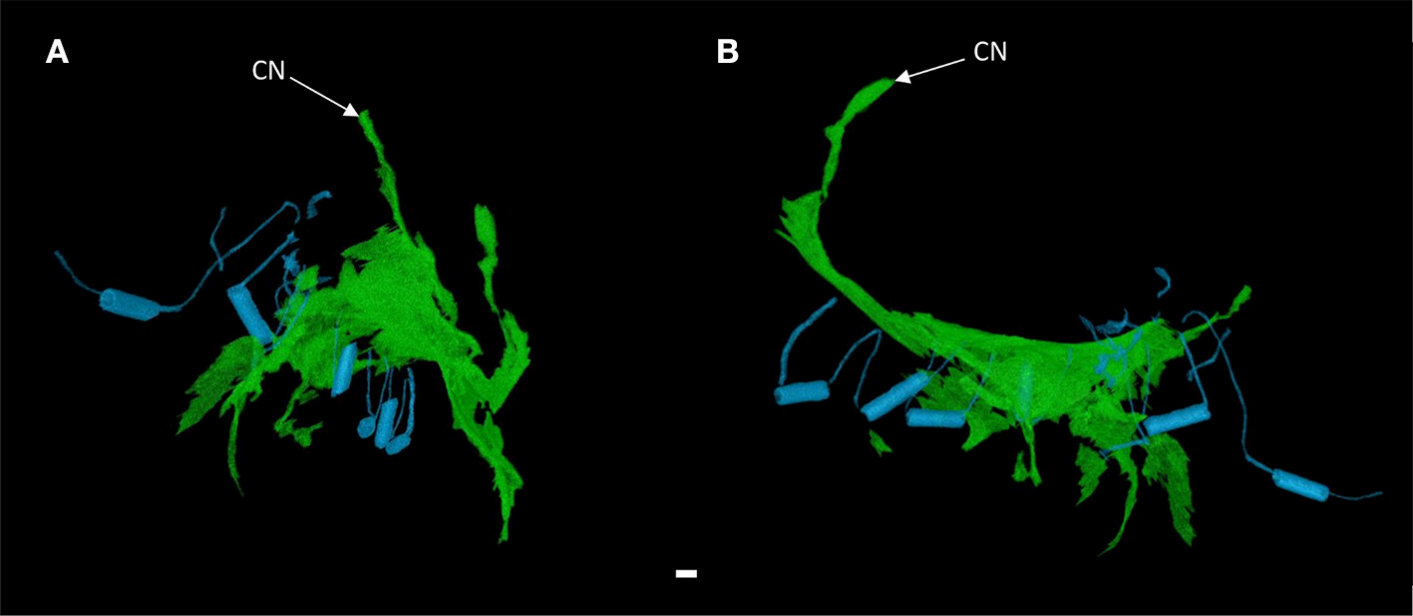
3-dimensional rendering of the atrioventricular conduction axis in a heart with atrioventricular septal defect (AVSD). Showing the conduction axis (green) and the surgically placed pledgets and sutures (blue) in anterior **(A)** and right lateral views **(B)**. Images derived from segmentation of micro-CT data. CN, compact atrioventricular node. Scale bar represents 1 mm.

Contrast enhancement permitted discrimination of multiple tissue types based on their differential attenuation of the x-ray source. As a result of differential iodine absorption; fat, myocardium, nodal tissues, and connective tissue presented decreasing voxel values respectively (Figure 3). The sinus node was located as a low attenuating (low voxel values) area in the intercaval region (Figures 1–3). The sinus node was seen to give off complex projections into the surrounding working myocardium, with a less pronounced paranodal region than that which is seen in the adult heart (Figure 3B). The compact atrioventricular node was notably displaced from its usual position at the apex of the triangle of Koch. The node was found in a posterior-inferior position anterior to the ostium of the coronary sinus at the point where the posterior-inferior rims of the muscular ventricular and atrial septa join, and was therefore housed in the inferior nodal triangle (Figures 1–4). The atrioventricular conduction axis (AVCA) and the proximal aspects of the right and left bundle branches could also be identified based on their differential attenuation (Figures 3D, 4). The conduction axis was seen to take a long and tortuous path across the crest of the muscular ventricular septum, with the proximal connection between the compact node and the axis appearing quite tenuous. The sinus node and atrioventricular compact node could be identified objectively in both the micro-CT image data (Figure 3) and the derived volume renderings (Figure 1). This is the first time the 3-dimensional disposition of the cardiac conduction system has been presented in a heart with AVSD.

It was apparent the heart had undergone attempted correctional surgery, namely the implantation of a surgical patch. This patch itself and the accompanying pledgets and suture lines could be identified in the micro-CT data (Figures 3C,D), and subsequently segmented and presented in 3-dimensions (Figures 2, 4). The patch had been attached superiorly at the free inferior margin of the atrial septum, which itself appeared hypoplastic. Inferiorly the pledgets and suture lines were placed deep into the right-hand aspect of the muscular ventricular septum. The sutures appeared to pass directly through the nodal tissue, particularly the right bundle branch (Figures 3D, 4).

**Figure 5:**
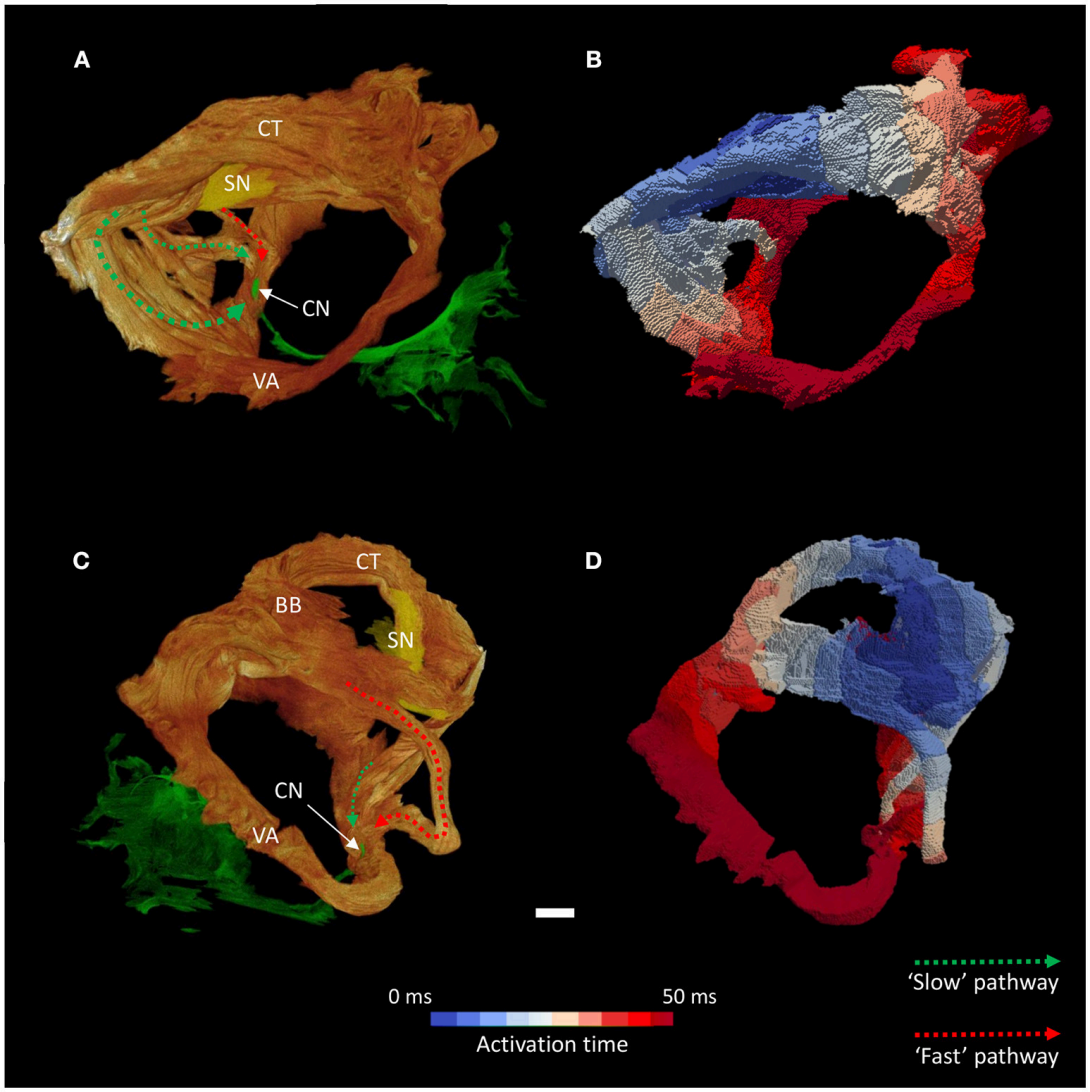
Inter-nodal conduction through the atrial muscle bundles I. Volume renderings of the atrial muscle bundles in right lateral **(A)** and left lateral **(C)** views, the normal location and direction of the “slow” pathway (green), and the septal aspect of the elongated “fast” pathway (red) are indicated by dotted arrows. **(B,D)** Display the corresponding electrical activation maps, showing excitation of the distal aspect of the region normally associated with the “slow” pathway precedes that of the “fast” pathway. See methods for modeling parameters. BB, Bachmann's bundle; CN, compact atrioventricular node; CT, terminal crest; SN, sinus node; VA, valve annulus. Scale bar represents 3 mm.

**Figure 6:**
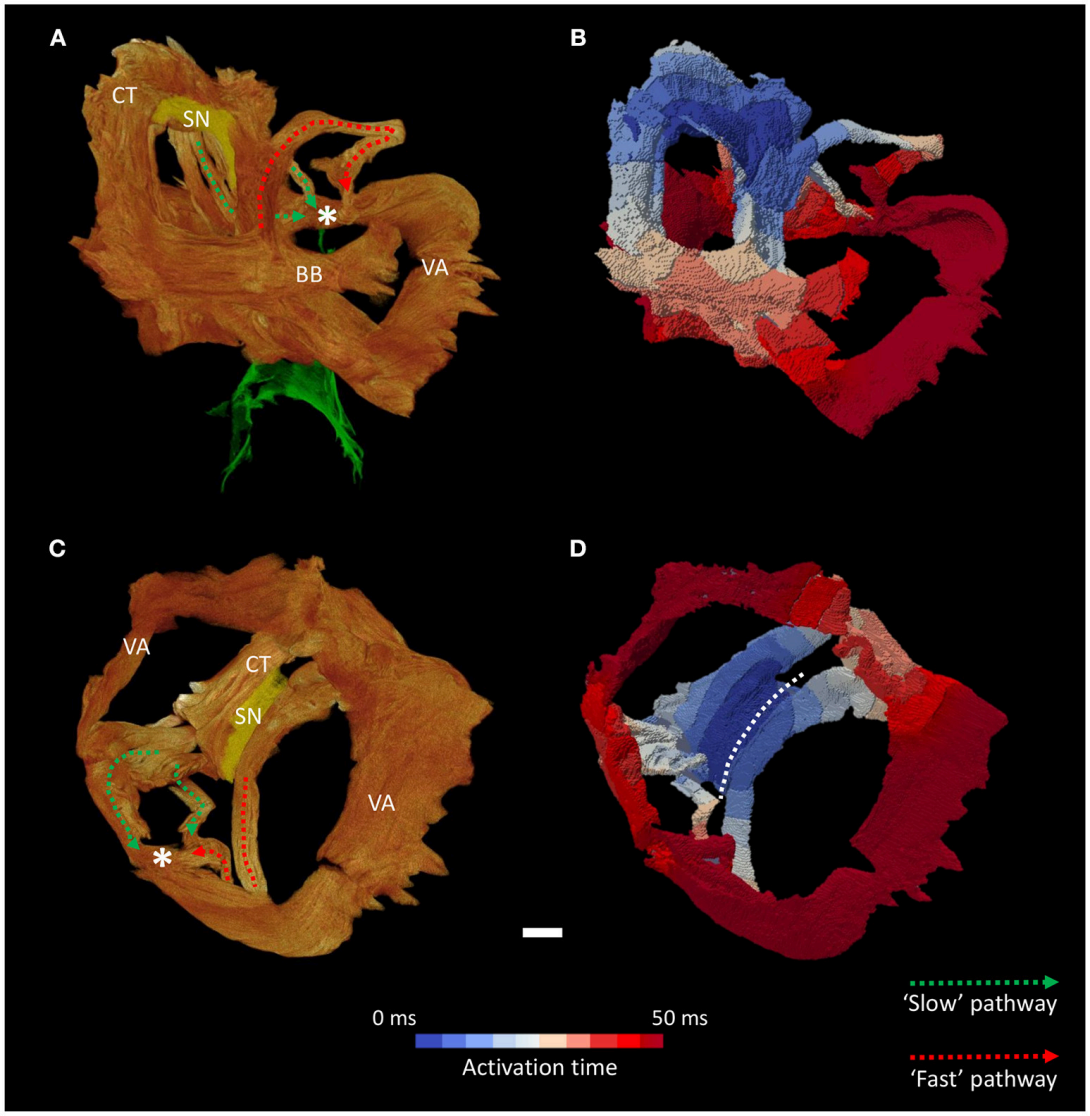
Inter-nodal conduction through the atrial muscle bundles II. Volume renderings of the atrial muscle bundles in anterior **(A)** and inferior **(C)** views, the normal location and direction of the “slow” pathway (green), and the septal aspect of the elongated “fast” pathway (red) are indicated by dotted arrows. **(B,D)** Display the corresponding electrical activation maps, showing excitation of the distal aspect of the region normally associated with the “slow” pathway precedes that of the “fast” pathway. See methods for modeling parameters. *Location of compact atrioventricular node; BB, Bachmann's bundle; CT, terminal crest; SN, sinus node; VA, valve annulus. Scale bar represents 3 mm.

The high resolution micro-CT data allowed the major muscle bundles of the atria to be identified and separated objectively based on their relatively parallel myocyte orientation. The terminal crest, Bachmann's bundle, common valve annulus and the bundles associated with the “slow” and “fast” pathways were segmented (Figures 5, 6). These bundles collectively formed a continuous “circuit” (Figures 5, 6). Note, the region normally associated with the distal aspect of the “fast” pathway showed a continuous connection with the common valve annulus and a distinct muscle bundle traversing the atrial septum (Figures 1B, 5, 6: red dotted lines). The mean orientation of the myocyte chains could be appreciated by following longitudinal features in volume renderings (Figures 1, 5, 6) and in the micro-CT image data (Figure 3B). Myocyte orientation analysis (see methods for details) confirmed that the mean orientation of the myocyte chains followed the long axis of these identified muscle bundles.

### Mathematical modeling

NB: When describing the modeling results in the AVSD heart we use the term “slow” and “fast” pathway based on the traditional identification of their anatomical position in the normal human heart, this is not a reflection of their conduction time.

We performed mathematical modeling of the wave of electrical depolarisation in both the isolated muscle bundles and the whole atria. We used a multi-cellular approach, with different models used for the sinus node, block zone, muscle bundles, and the atrial myocardium (see methods). The results of the myocyte orientation analysis were also incorporated into the models by allowing for faster conduction in the long axis of the myocytes than in the orthogonal directions (anisotropic conduction).

Activation maps (comprising isochrones) of the isolated muscle bundles showed that the fastest route to the atrioventricular compact node in this heart was via the “slow” pathway (Figures 5, 6). This is also clearly illustrated in Supplementary Video 1. The “fast” pathway connects with the compact node via the common valve annulus and a distinct septal muscle bundle traversing the atrial septum. Activation via this septal bundle arrived at the node 5–10 ms after the “slow” pathway (Figures 5, 6). The results therefore reflect a switch or flipping of the usual dual pathway physiology. The valve annulus provided the slowest route toward the compact node, and its activation was annihilated by stimulation via the “slow” and “fast” pathways in an anti-clockwise direction (Supplementary Video 1). These results were not affected by the presence or absence of the “block zone.”

**Figure 7:**
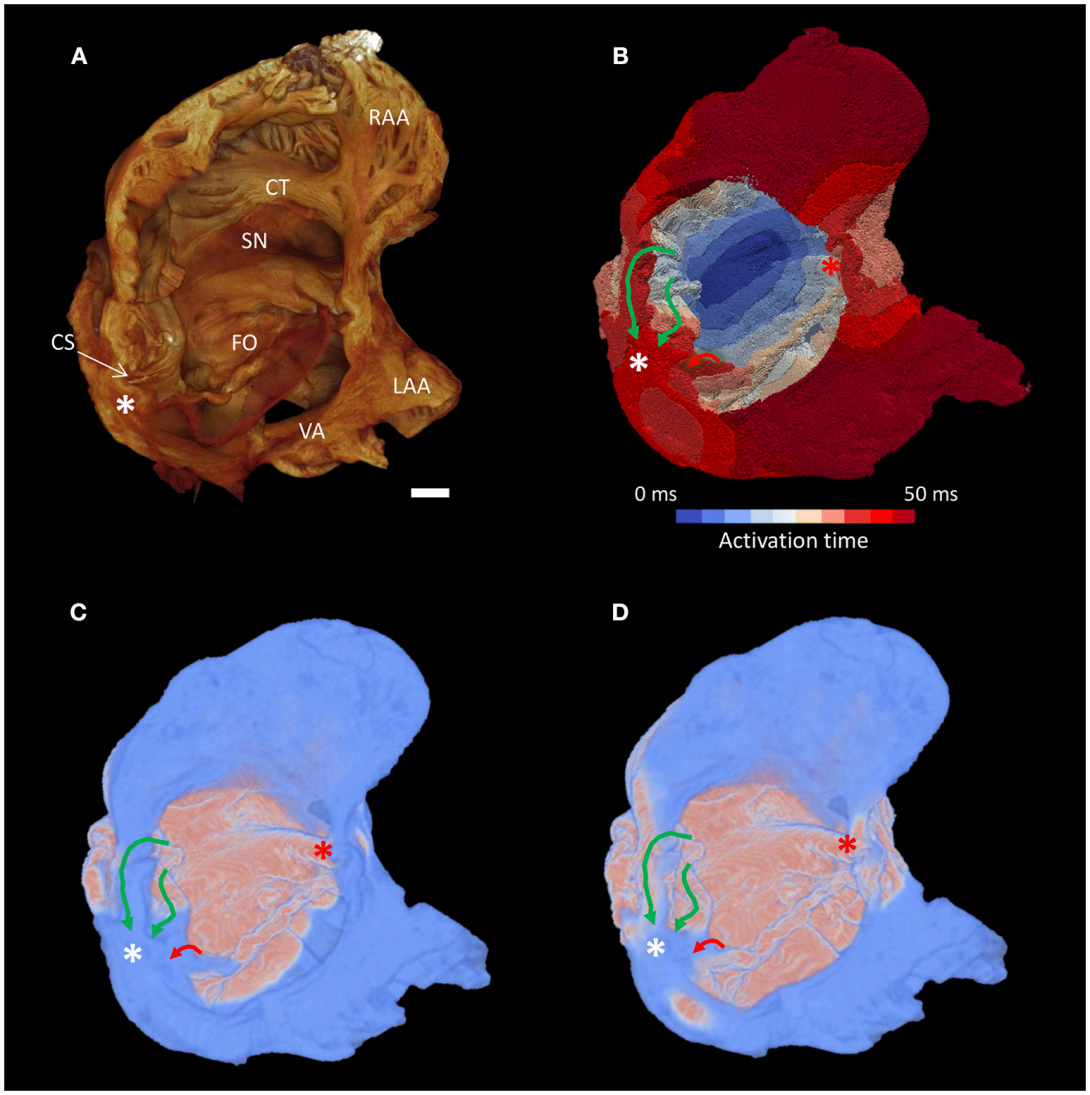
Preferential inter-nodal conduction via the “slow” pathway in the atria of a heart with AVSD. **(A)** Volume rendering of the atrial cavity viewed from the inferior-lateral position. **(B)** Corresponding isochrone electrical activation map, the normal location and direction of the “slow” pathway (green), and the septal aspect of the elongated “fast” pathway (red) are indicated by solid arrows. **(C,D)** Snapshots taken from the Supplementary Video 2 showing excitation of the distal aspect of the region normally associated with the “slow” pathway precedes that of the “fast” pathway, pink indicates activated myocardium, light blue indicates dormant myocardium. See methods for modeling parameters. White*, location of compact atrioventricular node in AVSD heart; Red*, approximate location of compact atrioventricular node in normal heart; CS, coronary sinus; CT, terminal crest; FO, fossa ovale; LAA, left atrial appendage; RAA, right atrial appendage; SN, sinus node; VA, valve annulus. Scale bar represents 3 mm.

**Figure 8:**
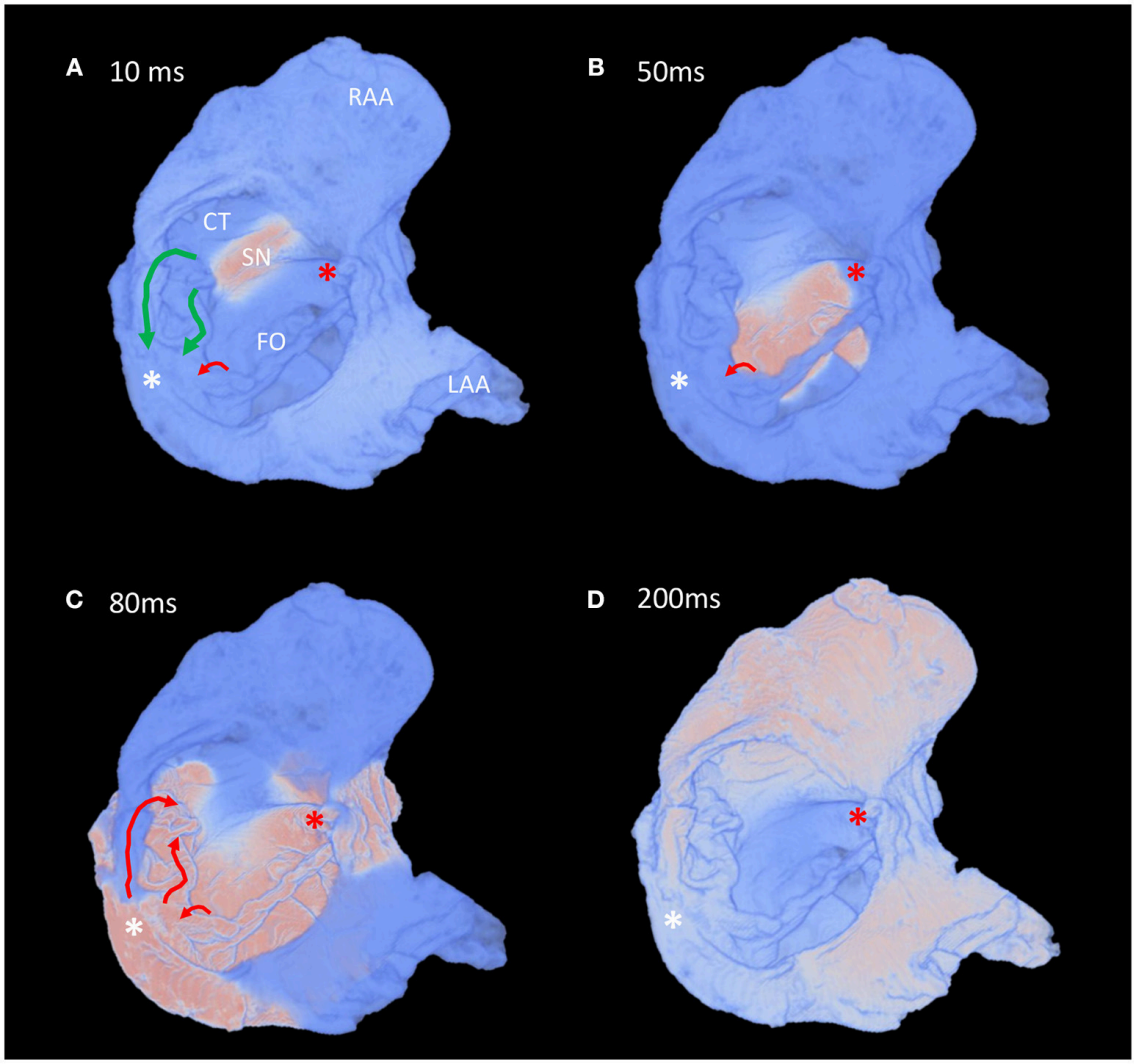
Fast pacing elicits retrograde conduction via the “slow” pathway in the atria of a heart with AVSD. **(A–D)** Time-lapse snapshots taken from the Supplementary Video 3 showing preferential inter-nodal conduction via the region normally associated with the “fast” pathway, and subsequent retrograde conduction up the “slow” pathway, during an atrial pacing protocol (s1-s2 interval 300 ms). Views are comparable to those presented in Figure 7. The normal location and direction of the “slow” pathway (green), and the septal aspect of the elongated “fast” pathway (red) are indicated by solid arrows. Pink indicates activated myocardium, light blue indicates dormant myocardium. See methods for modeling parameters. White*, location of compact atrioventricular node in AVSD heart; Red*, location of compact atrioventricular node in normal heart; CS, coronary sinus, CT, terminal crest; FO, fossa ovale; LAA, left atrial appendage; RAA, right atrial appendage; SN, sinus node.

Whole atrial modeling showed synchronous activation of the right and left atrial appendages and inter-atrial conduction preferentially via Bachmann's bundle. The results described above for the conspicuous muscle bundles were mirrored when modeling the whole atria, with the fastest route to the atrioventricular compact node seen to be via the “slow” pathway (Figure 7 and Supplementary Video 2). Figure 7 suggests the “fast” pathway would be the preferential pathway to the compact node were the node housed in the “normal” location (Figures 7B–D- red asterisk). Pacing of the whole atria with a 400 ms stimulus interval brought about normal sequential atrial activation. S2 intervals < 300 ms brought about atrial conduction block, with stimulus of the sinus node failing to elicit activation of the whole atria. In these scenarios the stimulus to atrial activation ratio approached 2:1. An S2 interval of 300 ms did, however, elicit atrial activation, but preferential activation of the compact node was no longer via the “slow” pathway. Preferential conductance and subsequent activation of the nodal region was provided by the “fast” pathway (Figures 8B,C). Nodal activation was followed by retrograde propagation up the “slow pathway” (Figure 8C). As a result the muscle bundles associated with “fast” pathway emerged from their refractory period before those of the “slow” pathway (Figure 8D). The pacing data presented in Figure 8 is presented as an animation in Supplementary Video 3.

## Discussion

In this study we show that contrast enhanced micro-CT is an effective non-destructive method for producing high-resolution, high-fidelity, 3-dimensional images of archived human hearts. From these images the 3-dimensional disposition of the cardiac conduction system and the complex arrangement of the myocyte chains can be resolved and quantified. To the best of our knowledge this is the first time such data has been presented for a heart with an AVSD. This high-resolution micro-anatomical data was then used to inform mathematical models of electrical activation, offering a potential stepwise change in the structural fidelity of such models. The resultant simulations are comparable to *in-vivo* clinical assessment of electrophysiology in AVSD patients, suggesting this is a viable technique for the investigation of arrhythmogenesis in congenitally malformed hearts *ex-vivo*.

### The competencies of micro-computed tomography

The nature of micro-CT data means that the morphological structure of this precious archived sample is forever preserved. This data is digital and thus will not degrade over time, and can be easily distributed and visualized using open source software. Thus, anatomists, surgeons, cardiologists, engineers, and teachers can easily make use of this new information.

The micro-CT data allowed for fast diagnosis and classification of the defect. Virtual histology (Figure 3) and virtual dissection (Figures 1, 2) can be performed rapidly and non-destructively in an infinite number of planes. This has clear advantages over traditional destructive, laborious, and error prone techniques such as histology and blunt dissection. As described previously (Stephenson et al., 2012, 2017), contrast enhancement allowed the specialized cells of the cardiac conduction system to be resolved independent of the surrounding working myocardium and connective tissue. The disposition of the nodal tissues described in the present study is consistent with previous anatomical accounts of hearts with AVSD using traditional techniques (Anderson et al., 2000). Consistent with previous accounts in the adult human heart (Boyett et al., 2000; Sánchez-Quintana et al., 2005; Fedorov et al., 2010; Stephenson et al., 2017), the sinus node was irregular in shape and occupied a large portion of the inter-caval region, and was seen to give off complex projections into the surrounding myocardium. The sinus node in the AVSD heart did however appear to have a less pronounced paranodal area compared with the adult (Chandler et al., 2011; Stephenson et al., 2017). The nature of the defect and the posterior-inferior displacement of the compact atrioventricular node made for an elongated AVCA, this has been described previously, and is thought to contribute to the prevalence of atrioventricular node block in these patients (Feldt et al., 1970; Anderson et al., 2000, 2008).

In the present study, and previously (Aslanidi et al., 2012; Ni et al., 2013; Stephenson et al., 2017), we have demonstrated how myocyte orientation can be extracted from high-resolution micro-CT data. Extraction of myocyte orientation is imperative to allow accurate modeling of cardiac electrical activation. Conduction is known to be faster along a cardiomyocyte chain's longitudinal axis than across its short axis (Spach and Kootsey, 1983). The course of the cardiomyocyte chains and their aggregation into distinguishable muscle bundles, therefore, plays a crucial role in inter-nodal conduction. This is highlighted in modeling data presented in the current study (Figures 5–8), and illustrates the importance of the whole heart high-resolution data presented here.

### Substrates for arrhythmogenesis in a heart with AVSD

NB: When describing the modeling results in the AVSD heart we use the term “slow” and “fast” pathway based on their anatomical position in the normal human heart, this is not a reflection of their conduction time.

The simulations of atrial activation produced in the present study show preferential activation of the compact atrioventricular node via the region normally associated with the “slow” pathway (Figures 5–7). This flipping of the dual pathway physiology is consistent with previous *in-vivo* three-dimensional electroanatomic mapping studies, in which the slowest pathway was located superior to AVCA, while the fastest pathway was identified posterior-inferior to the compact node (Khairy et al., 2007; Khairy and Balaji, 2009). The arrangement is best observed in the right hand and left hand views shown in Figure 5. This phenomenon is not surprising considering the displacement of the compact node implies a physical shortening of the “slow” pathway and a concomitant lengthening of the “fast” pathway (Figure 1). In this regard, we show how the distal aspect of the region normally associated with the “fast” pathway is continuous with a septal muscle bundle and the common valve annulus. Contrary to the findings of Khairy and associates (Khairy et al., 2007), our modeling data suggests conduction along the leftward aspect of the valve annulus is slow and is annihilated by the “slow” pathway (Supplementary Videos 1, 2). The preferential route for the “fast” pathway is, therefore, via the septal region. Our data therefore supports the suggestion of Waldo and associates that the middle and anterior (corresponding to the “fast” pathway) inter-nodal pathways may become distorted or modified due to the septal defect (Waldo et al., 1973).

The area anterior-inferior to the fossa ovale, which in the normal heart houses a distinct muscle bundle and the region of the inferior nodal extension, was seen to be hypoplastic and damaged by the suture placement associated with the patch implantation. This suggests, in this instance, that this region is not a viable route, and that inter-nodal conduction runs posterior-superior to the fossa ovale via a septal bundle. Patch placement in the ventricle can also have functional implications. It is hindered by the need to attach the patch to the right hand aspect of the muscular ventricular septum in order to close the defect. Fournier and associates observed right bundle branch block in 19 of 25 postoperative patients (Fournier et al., 1986). Right bundle branch block has historically been a problem in AVSD patients and the necessary placement of pledgets and sutures in the current sample demonstrate the challenge facing the reconstructive surgical team (Figures 3D, 4). In this regard micro-CT data has potential implications in the planning of corrective surgery and ablation therapy, pathological reporting, and for investigations into the history of surgical approaches.

Retrograde atrial activation via the fastest conducting pathway has been observed previously in AVSD patients (Khairy et al., 2007), and in this case cryomapping of the slowest conducting pathway can relieve the accompanying atrioventricular re-entry tachycardia (AVNRT). In the present study atrial pacing using a S2 interval of 300 ms elicited whole atrial activation, but preferential activation of the compact node was no longer via the “slow” pathway. Preferential conductance and subsequent activation of the nodal region was provided by the “fast” pathway (Figures 8B,C and Supplementary Video 3). Nodal activation was then followed by retrograde propagation up the “slow” pathway (Figure 8C and Supplementary Video 3). As a result the muscle bundles associated with “fast” pathway were seen to leave their refractory period before those of the “slow” pathway (Figure 8D and Supplementary Video 3). In this setting the dual pathway physiology therefore becomes desynchronised which could perpetuate both typical and atypical AVNRTs. This finding also provides reasoning for other electrical disturbances observed clinically, such as slow inter-nodal conduction, atrial fibrillation and atrioventricular block (Daliento et al., 1991; Khairy et al., 2006). Furthermore, the data provides evidence to support ablation of the slowest conducting pathways in this setting.

Our findings suggest that displacement of the compact atrioventricular node and the accompanying structural modification of the dual pathway physiology provides morphological substrates for arrhythmogenesis in hearts with AVSD.

### Future perspectives

The methodologies and concepts presented in the current study provide the opportunity to investigate, and potentially resolve, controversies regarding the anatomical substrates for inter-nodal conduction (Anderson et al., 1981; Sanchez-Quintana et al., 1997; Hucker et al., 2008). Future studies using this dataset could include atrio-ventricular and whole heart modeling to investigate substrates and ablation strategies for ventricular tachycardia, atrioventricular block, and bundle branch block, all of which are frequently observed in this defect (Daliento et al., 1991; Khairy, 2008; Khairy and Balaji, 2009). Furthermore, there are many other cardiac congenital malformations that are associated with specific electrical disturbances and arrhythmias. This study is a “proof of concept,” opening the door for wide-scale investigation of arrhythmogenesis by topographical micro-anatomy combined with numerical simulation of electrical activity in the congenitally malformed heart.

## Study limitations

We recognize that this study lacks an age-matched healthy control to validate our findings, but such a sample would be extremely difficult to obtain. The major limitation of this study is the need to downsample the high-resolution information-rich micro-CT data into a form which is computationally manageable. The large file size, in this case ~10 GB, and the fine structural details, make the integration of such data into mathematical models, computationally and theoretically difficult. This, however, highlights a new research challenge for the modeling and engineering community. While providing new challenges, high resolution micro-CT data does provide a step change in the quality of structural geometries available to groups working on mathematical models of cardiac depolarisation.

## Data availability

The datasets for this manuscript are not publicly available because: this patient data is sensitive and ethical approval is acquired on an individual basis. Requests to access the datasets should be directed to Dr. Robert Stanley Stephenson, email: robert.stephenson@clin.au.dk.

## Author contributions

JJ, CJ, and RG acquisition of ethical approval for the study. JJ and RS sample preparation and RS, JJ, and TL data acquisition. JR-N, RS, and HZ data analysis and production of figures. RS writing and JR-N, CJ, RG, JZ, HZ, and JJ editing of manuscript.

### Conflict of interest statement

The authors declare that the research was conducted in the absence of any commercial or financial relationships that could be construed as a potential conflict of interest.
