# Supplementary material for: Morphological Substrates for Atrial Arrhythmogenesis in a Heart With Atrioventricular Septal Defect
Source: Front Physiol. 2018 Aug 23;9:1071. doi: 10.3389/fphys.2018.01071 (PMC6115687; doi:10.3389/fphys.2018.01071)
Supplement: Supplementary file 4 [file Data_Sheet_1.docx]

**Morphological substrates for atrial arrhythmogenesis in a heart with atrioventricular septal defect**

Robert S. Stephenson^1*^, Jack Rowley-Nobel^2^, Caroline B. Jones^3^, Rafael Guerrero^4^, Tristan Lowe^5^, Jichao Zhao^6^, Henggui Zhang^2^, Jonathan C. Jarvis^7^.

1. Comparative Medicine Lab, Institute of Clinical Medicine, Aarhus University, Aarhus, Denmark.
2. The School of Physics and Astronomy, University of Manchester, Manchester, UK.
3. Department of Cardiology, Alder Hey Children’s Hospital, Liverpool, UK.
4. Department of Cardiac Surgery, Alder Hey Children’s Hospital, Liverpool, UK.
5. Manchester X-ray Imaging Facility, Photon Science Institute, University of Manchester, Manchester, UK.
6. Auckland Bioengineering Institute, Auckland University, Auckland, New Zealand.
7. School of Sport and Exercise Sciences, Liverpool John Moores University, Liverpool, UK.

**Correspondence:**

Dr Robert Stanley Stephenson

Robert.stephenson@clin.au.dk

**Supplementary material**

**Video legends**

**Supplementary video 1.** Inter-nodal conduction through the atrial muscle bundles. Showing excitation of the distal aspect of the region normally associated with the ‘slow’ pathway precedes that of the ‘fast’ pathway. The septal aspect of the elongated ‘fast pathway’ is indicated by the red arrow. The simulation is viewed superiorly. Pink indicates activated myocardium, light blue indicates dormant myocardium. Simulation time period equals 180 milliseconds. See methods for modelling parameters. White*- location of compact atrioventricular node, CS- coronary sinus, VA- valve annulus.

**Supplementary video 2.** Preferential inter-nodal conduction via the ‘slow’ pathway in the whole atria of a heart with AVSD. Showing excitation of the distal aspect of the region normally associated with the ‘slow’ pathway precedes that of the ‘fast’ pathway. The septal aspect of the elongated ‘fast pathway’ is indicated by the red arrow. The simulation is viewed inferiorly. Pink indicates activated myocardium, light blue indicates dormant myocardium. Simulation time period equals 112 milliseconds. See methods for modelling parameters. White*- location of compact atrioventricular node, LAA- left atrial appendage, RAA- right atrial appendage, SN- sinus node.

**Supplementary video 3.** Fast pacing elicits retrograde conduction via the ‘slow’ pathway in the atria of a heart with AVSD. Showing preferential inter-nodal conduction via the region normally associated with the ‘fast’ pathway, and subsequent retrograde conduction up the ‘slow’ pathway, during an atrial pacing protocol (s1-s2 interval 300 ms). The septal region of the elongated ‘fast’ pathway is indicated by the red arrow. The simulation is viewed inferiorly. Pink indicates activated myocardium, light blue indicates dormant myocardium. Simulation time period equals 1000 milliseconds. See methods for modelling parameters. White*- location of compact atrioventricular node, LAA- left atrial appendage, RAA- right atrial appendage, SN- sinus node.
